# The Role of Ni in Stabilizing BaZrO_3_ Surfaces at Ni/BaZrO_3_ Interfaces: A Density Functional Theory Analysis

**DOI:** 10.1021/acs.langmuir.5c06802

**Published:** 2026-07-08

**Authors:** Maxim Shishkin, Atsushi Ishikawa

**Affiliations:** Department of Transdisciplinary Science and Engineering, School of Environment and Society, 13290 Institute of Science Tokyo, 2-12-1 Ookayama, Meguro-Ku, Tokyo 152-8552, Japan

## Abstract

Using previous computational findings on the stability of surface terminations of BaZrO_3_ as a starting point, we discuss how adsorbed Ni could introduce additional stable terminations of BaZrO_3_. As a benchmark, we show that BaO(001) is indeed the most stable termination of pure BaZrO_3_, whereas (011) and (111) terminations have higher surface energies, in agreement with previous studies. We also discuss the underlying reasons for the greater stability of (001) versus (011) and (111) terminations and show that the high coordination numbers of Zr and Ba on (001) surfaces are key factors responsible for the lower free energy of (001) terminations. With regard to Ni cluster adsorption, we demonstrate that the (011) termination could be substantially stabilized, particularly due to the presence of additional oxygens at the Ni/BaZrO_3_ interface, which could occupy otherwise empty sites of a stoichiometric slab of BaZrO_3_ with (011) terminations. In contrast, the Ni/BaO(001) termination tends to be less stable (depending on coverage and the amount of adsorbed Ni) due to the less favorable introduction of oxygens at the interface with Ni. Using calculated phase diagrams and Wulff constructions, we comment on the percentage of (011) terminations in crystal shapes. We also discuss the impact of Y doping on the relative stabilization of BaZrO_3_ surfaces with adsorbed Ni, proposing that conclusions similar to those for undoped Ni/BaZrO_3_ could be drawn. Our work shows that, in addition to the models of Ni/BaO(001) interfaces, the models of Ni mounted on the (011) termination of BaZrO_3_ should be considered for adequate modeling of reactions at the metal/oxide boundaries, which are essential for electrochemical processes in fuel cells and electrolyzers.

## Graphical abstract

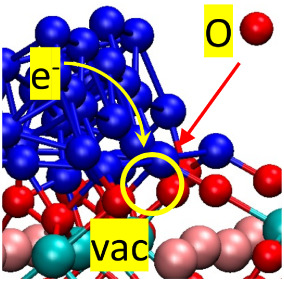

## 1 Introduction

Metal/oxide interfaces are widely employed as structures that catalyze chemical and electrochemical reactions, finding applications in numerous fields such as fuel cells,^1^ water electrolysis,^2^ ammonia decomposition,^3^ hydrocarbon reforming,^4^ and CO_2_ reduction.^5^ Out of the various oxides that act as metal supports in such reactions, perovskite materials stand out as structures with a wide range of possible electronic and/or ionic conductivities, as well as favorable mechanical and thermal characteristics.^6–8^ In particular, BaZrO_3_ perovskite and its more complex derivatives (e.g., doped structures with partially replaced Ba or Zr atoms) combined with Ni catalysts are commonly used as anodes of proton-conducting fuel cells (PCFCs) due to the favorable catalytic properties of Ni and the high protonic conductivity and stability of BaZrO_3_-based structures.^9–11^ The high catalytic activity of Ni accounts for fuel activation (e.g., dissociation of hydrogen molecules) with a subsequent spillover reaction from Ni to BaZrO_3_-based structure and proton migration into the perovskite bulk (extended Ni clusters should be differentiated from possible Ni doping, as extended structures also account for the electrical conductivity of the anode materials, unlike doping). Accurate analysis of such reactions at the boundary between Ni and doped BaZrO_3_ heavily relies on adequate models of the combined configurations of metals and oxides, including the types of surfaces formed, dopant segregation, and boundary defect formation.

The surface energies of BaZrO_3_ have been studied previously using *ab initio* thermodynamics approach for the purpose of determining the most stable surfaces.^12^ Analyzing terminations with low Miller indices, namely (001) and (011), with various types of atomic configurations of the surfaces of employed slabs, the authors of ref. 12 established that the BaO(001) termination has the lowest free energy of formation (the stoichiometry of the topmost layer (BaO) is used as a surface notation). The ZrO2(001) termination has been found to be unstable with respect to the precipitation of a constituent oxide (i.e., ZrO_2_), and its formation has been ruled out for any possible ambient conditions (for surface notation, we use large indices for the number of atoms in the formula unit (e.g., in ZrO_2_) to differentiate from the bulk units where numbers are provided as subscripts). Moreover, the (011) terminations have been found to be less stable than the BaO(001) termination too.^12^ Additionally, the electronic and structural properties of BaZrO_3_ have also been studied in subsequent works.^13–15^

In view of the established high stability of the BaO(001) termination, subsequent works on the analysis of reactions on the surface of the metal/BaZrO_3_ interface have been performed using slabs where BaZrO_3_ is exposed to the gas phase by the BaO(001) termination.^16–18^ The metal clusters (e.g., Pd, Ni, Pt, etc.) have been designed in such a way that the metal faces the vacuum of the slab via the (111) termination, which has lower surface energy than the (001) and (011) terminations of the fcc lattice. Using such models, the reactions of proton or oxygen migration from the metal catalyst to the proton-conducting BaZrO_3_ (spillover reactions) have been studied using density functional theory (DFT) calculations.^16–18^ Spillover reactions, in addition to the migration of protons in the BaZrO_3_ bulk, studied in several computational works, are important steps in the overall process of PCFC operation and should be modeled using reliable interface structures.

The {001} surfaces have also been observed in experimental works on the analysis of BaZrO_3_ particles. For instance, cubic nanoparticles with the {001} family of surface terminations have been synthesized and observed experimentally by Chamorro et al.,^19^ corroborating computational conclusions about the exceptional stability of {001} surfaces of BaZrO_3_. On the other hand, Kanie et al. demonstrated that other terminations of BaZrO_3_ might be present in synthesized particles of about 7 μm in diameter if precursors containing Cl, N, and CH_3_ species are used as Zr sources.^20^ Depending on the amount of Zr introduced during BaZrO_3_ synthesis, they have shown that fine particles of various shapes could be observed, including particles with clearly present {011} terminations.^20^ This introduction of {011} terminations has been attributed to stabilizing adsorbates on the perovskite surface, although the details about the types of such surface adsorbates, as well as the mechanisms of stabilization of {011} terminations, have not been discussed. Additionally, BaZrO_3_ nanocrystals with {001}/{011} facets have also been observed in the work of Meng et al.,^21^ who applied a solvothermal method for their synthesis using the same type of Zr-containing precursors with Cl as those used in the work of Kanie et al.^20^ Thus, several experimental works indicate that, in addition to {001} terminations, which were found to be the most stable by DFT analysis, synthesized particles of BaZrO_3_ might also feature {011} terminations, although the underlying reasons for the introduction of {011} have not been clarified in sufficient detail.

In this work, we aim to analyze the impact of adsorbates on BaZrO_3_ surface stability. However, instead of possible surface species that can be introduced as byproducts of Zr-containing precursors (e.g., those with Cl and N), we wish to analyze the impact of Ni atomic clusters, viewed as adsorbates on the surface of perovskite. Ni is added to the surface of BaZrO_3_ to account for electrical conductivity and transfer of electronic charge, in addition to acting as a catalyst for hydrogen adsorption with subsequent spillover to the perovskite surface and bulk. The Ni clusters could affect the stability of the perovskite surface, similar to the precursor adsorbates, as has been shown in the experiments described in refs. 20,21 . As a result, the introduction of other surface terminations, in addition to BaO(001), should be considered in view of their potential stabilization by surface Ni.

This paper is organized as follows. The details of the employed methodology are discussed in Section 2. In Section 3.1, we present the analysis of the stability of surface terminations of BaZrO_3_ for benchmarking the previous literature results. In addition to {001} and {011} surfaces, we also provide a comparison with the stability of {111} surfaces, which have not been studied previously using *ab initio* thermodynamics analysis.^12^ In Section 3.2, we proceed to the analysis of the impact of Ni on BaZrO_3_ terminations and construct the respective phase diagrams. The role of doping via partial substitution of Zr by Y in the relative stabilization of perovskite surfaces is discussed next. The conclusion (Section 4) summarizes our findings on the role of Ni in the stabilization of BaZrO_3_ surfaces.

## 2 Computational Methods

### 2.1 Details of Calculation

VASP (Vienna *ab initio* Simulation Package) was employed for calculations of energetics, electronic structure, and structural properties of the studied configurations (we used version 6.4.3).^22^ The PBE (Perdew–Burke–Ernzerhof) functional has been applied for the description of exchange-correlation effects,^23^ and the PAW (Projector Augmented Wave) approach has been used for calculations of interactions of electrons with the cores of atomic nuclei in the studied structures.^24,25^ The PBE functional has been employed similarly to previous works on BaZrO_3_.^12^ The van der Waals corrections have not been applied, as improvement of critical properties such as cohesive energies, lattice constants, and bandgaps is not necessarily obtained for materials such as Ni.^26^

Hubbard corrections (DFT + *U* method^27^) are usually not applied to BaZrO_3_, as PBE calculations predict the presence of a bandgap for this insulating material. Hybrid functionals, particularly of HSE06 type,^28^ which are most suitable for solids, could also be a possible alternative to PBE due to the generally higher precision of predicted properties, such as bandgaps, energies of formation, etc. However, a much higher computational cost as compared to PBE calculations makes the HSE06 approach less suitable for the analysis of large and complex atomic systems like those analyzed herein. In the Supporting Information, we also comment on the accuracy of HSE06 calculations in the evaluation of formation energies of constituent oxides, analyzed in this work.

Accurate evaluation of the total energies of metals and their respective oxides (e.g., Ba, Zr, and BaO, ZrO_2_) is a challenging task, and even advanced functionals (e.g., hybrid and beyond) are generally unable to provide a good agreement of energies of formation with available experimental data (see discussion in the Supporting Information). For this reason, we rely on an empirical correction of calculated energetics, as proposed in previous works.^12^ In the Supporting Information, we describe how the correction of the total energy of an oxygen molecule should be introduced in order to gain values of the energies of formation of the constituent oxides (BaO and ZrO_2_) in closer agreement with experimental data. In this work, we employ this empirically corrected energy of the oxygen molecule for all quantities required for phase diagram construction and vacancy formation description. Additionally, we relied on empirical data for the evaluation of free energies (e.g., the entropy of H_2_ and H_2_O molecules), also provided in the Supporting Information.

Electronic structure optimization was performed using a conjugate gradient method. The cutoff energies of 600 eV have been used for all structures analyzed in our work (the bulk structures of BaZrO_3_ as well as constituent oxides (BaO, BaO_2_, and ZrO_2_), the bulk structures of Ba and Zr metals, and also all employed slabs). The chosen value of the cutoff provides well-converged energies of formation according to our tests; e.g., the energy of ZrO_2_ formation is found to converge below 0.001 eV/unit. For systems without added Ni, we have been using nonspin-polarized PBE calculations, whereas for Ni-containing models, spin-polarized DFT has been employed.^29^

Structural optimization has been performed so that the Hellmann–Feynman forces do not exceed 0.03 eV/Å. The convergence of total energies with respect to the Monkhorst–Pack *k*-point sampling^30^ has been checked for all studied bulk structures. For the slab configurations, we have employed the *k*-point mesh with a density of at least 0.4 points/Å in horizontal planes (the Γ-point has been used in the vertical dimension). The *k*-point mesh in horizontal planes has been tested with respect to the convergence of calculated quantities. We have also tested the convergence of calculated energies with respect to the thickness of atomic slabs and the size of the chosen vacuum layer, adopting sufficiently long atomic slabs and a large vacuum size. Moreover, we have tested that the inclusion of dipole corrections also yields well-converged values of the calculated total energies of the employed slabs.

### 2.2 *Ab Initio* Thermodynamics and Crystal Shapes

The details of *ab initio* thermodynamics approach,^31–34^ including specific formulations for perovskite materials,^35–39^ have been discussed in previous seminal papers. The formalism is sufficiently established in the literature, so we comment only briefly about key quantities that are used for the description of the energy of surface terminations.

The free energy of a surface of a material that consists of three elements (which is the case of BaZrO_3_) is a function of the chemical potentials of only two elements, as only two chemical potentials are independent quantities.^35–39^ In this work, we choose the chemical potentials of O and Zr as independent variables. The free energy is defined as

1 $$\gamma =\frac{1}{2}\left(E_{slab}-N_{Ba}E_{BaZrO_{3}}^{bulk}-\Delta N_{Zr}\left(E_{Zr}+\Delta \mu_{Zr}\right)-\Delta N_{O}\left(E_{O}+\Delta \mu_{O}\right)\right)$$

where *E*_*slab*_ is the energy of a symmetric slab, which has two identical terminations; *N*_*Ba*_ is the total number of Ba atoms in the slab; $E_{BaZrO_{3}}^{bulk}$ is the energy of the BaZrO_3_ bulk unit; Δ*N*_Zr_ and Δ*N*_*O*_ are the differences in the numbers of Zr and O atoms in the slab and combined bulk units, equal to $N_{Zr}-N_{Ba}$ and $N_{O}-3\times N_{Ba}$ , respectively. *E*_*Zr*_ and *E*_*O*_ are evaluated as the energies of Zr in Zr bulk and O in the oxygen molecule, respectively. The free energy of the surface is a function of variations of the chemical potentials Δ*μ_Zr_* and Δμ_*O*_.

Although the energy of Zr could be calculated in a straightforward manner as the energy of an ion in Zr bulk, the energy of an oxygen atom (*E*_*O*_) usually requires corrections due to the known inability of DFT to provide an accurate value for the energy of an oxygen molecule. In this work, we rely on a common procedure for correcting the total energy of an oxygen molecule based on empirical data for the energy of formation of Zr oxide.^12^ The correction to the energy of the oxygen molecule is obtained from the experimental energy of formation of the monoclinic phase of zirconia (Supporting Information provides further details), which corresponds to the ground state at low temperatures (below 1170 °C). The obtained corrected energy of the oxygen molecule is −8.78 eV, which is significantly higher than the value of the total energy of O_2_ in a triplet state, as provided by PBE (−9.86 eV).

The values of the free surface energies of studied terminations are usually normalized by the surface area of their respective slabs for comparison. However, the free energies, as of eq 1 allow for comparison of surface stability with respect to metals (e.g., Zr, Ba) and oxygen gas molecules. In addition, stability with respect to the precipitation of constituent oxides should also be checked. In the case of BaZrO_3_, three such oxides are BaO, BaO_2_, and monoclinic ZrO_2_. The stability condition that should be fulfilled with respect to the precipitation of ZrO_2_:

2 $$\Delta \mu_{Zr}+2\Delta \mu_{O}<E({\mathrm{ZrO}}_{2})-E\left(\mathrm{Zr}\right)-h\left(O_{2}\right)$$

where *h*(*O*_2_) is a corrected value of the enthalpy of an oxygen molecule. Similarly, for BaO and BaO_2_ oxides:

$$\Delta \mu_{Zr}+2\Delta \mu_{O}<(E\left({\mathrm{BaZrO}}_{3}\right)-E\left(\mathrm{Ba}\right)-E\left(\mathrm{Zr}\right)-\frac{3}{2}h\left(O_{2}\right))-\left(E\left(\mathrm{BaO}\right)-E\left(\mathrm{Ba}\right)-\frac{1}{2}h\left(O_{2}\right)\right)$$ 3

$$\Delta \mu_{Zr}+\Delta \mu_{O}<(E\left({\mathrm{BaZrO}}_{3}\right)-E\left(\mathrm{Ba}\right)-E\left(\mathrm{Zr}\right)-\frac{3}{2}h\left(O_{2}\right))-(E({\mathrm{BaO}}_{2})-E\left(\mathrm{Ba}\right)-h\left(O_{2}\right))$$ 4

Additional requirement that should be satisfied for the variations of chemical potentials is the stability of BaZrO_3_ bulk:

5 $$\Delta \mu_{Zr}+3\Delta \mu_{O}<E({\mathrm{BaZrO}}_{3})-E\left(\mathrm{Ba}\right)-E\left(\mathrm{Zr}\right)-\frac{3}{2}h\left(O_{2}\right)$$

Based on the conditions provided in eqs 2–5, in addition to the requirement of positive free energies for all studied surfaces (eq 1), the phase diagram could be constructed.

Complementary to phase diagrams, the crystal shapes obtained via the Wulff approach could also be used for the determination of a relative percentage of various observable surface terminations. Crystal shapes could be constructed using the following condition for the boundary planes that overlap the volume around a chosen center point:

6 $$d_{hkl}=\theta \times \gamma_{hkl}$$

In eq 6, γ_*hkl*_ is the free energy of a surface with Miller indices (*hkl*) (defined in eq 1), *d*_*hkl*_ is the distance between the center point and the (*hkl*) plane, and θ is a chosen constant. The value of θ is not critical if only a crystal shape is of interest, as it defines the size (or diameter) of the chosen crystal shape.

## 3 Results and Discussion

### 3.1 Analysis of BaZrO_3_

#### 3.1.1 Surface Stability of BaZrO_3_

The study, based on the methodology discussed in Section 2.2, has been performed for BaZrO_3_ terminations in the past.^12^ Various possible terminations of (001) and (011) surfaces have been included in the comparative analysis of surface stability.^12^ In this work, we choose to analyze only the surface terminations where the numbers of atoms of all types (Ba, Zr, and O) in the respective symmetric slabs are subject to the condition

7 $$N_{Ba}:N_{Zr}:N_{O}=1:1:3$$

or:

8 $$N_{Ba}:N_{Zr}:N_{O}=M:L:\left(M+2L\right)$$

where *M* and *L* are integer numbers (in eq 8 *M* is not equal to *L*). Slabs for which eq 7 is satisfied are termed stoichiometric. For slabs where eq 8 is valid, the numbers of atoms can be combined in a sum of multiples of BaO and ZrO_2_ units with coefficients *M* and *L*. These slabs are termed as combined stoichiometric, as their stoichiometry can be viewed as a combined stoichiometry of ZrO_2_ and BaO oxides.

The slabs subject to eqs 7 or 8 allow for the oxidation states of all atoms (Ba^2+^, Zr^4+^, and O^2–^) to be the same as in the bulk oxides (BaZrO_3_, BaO, ZrO_2_). For this reason, the surface stability of such slabs tends to be higher than in “nonstoichiometric” slab, where the number of constituent atoms does not allow for bulk-like oxidation states of all atoms in the slab.

The previous work on the analysis of surface stability of BaZrO_3_ corroborates the conjecture that only slabs, subject to eqs 7 or 8, are the most stable, finding that BaO and ZrO_2_ terminations of the (001) surface are the only two candidates present in the final phase diagram.^12^ These correspond to the slabs with combined stoichiometry (eq 8), which possibly accounts for their exceptional stability. It should be noted that a stoichiometric slab (according to eq 7) with a (011) surface has also been studied in ref. 12 (it was denoted as O(011)). In this work, however, we denote this termination as BaZrO2(011), taking into account all atoms of the respective termination, not only the outermost oxygens. Calculations in ref. 12 also revealed that the surface energy of BaZrO2(011) is always higher than the two terminations of the (001) surface for all possible values of the chemical potentials of constituent atoms. In this work, we aim to benchmark this finding, and in addition to ref 12, we also analyzed the slabs with (111) terminations, particularly those subject to eqs 7 or 8.

In Figure 1, we provide our constructed phase diagram for surface terminations of BaZrO_3_. The corresponding chemical potentials of oxygen in a hydrogen atmosphere, in the range of PCFC operating temperatures (400–700 °C), which also covers the synthesis temperature of Ni/BaZrO_3_,^9^ are indicated by the blue broken lines. Supporting Information provides the details on how the respective values have been obtained. The slabs presented in Figure 1 have been designed using the optimized bulk of BaZrO_3_ (the bulk cell could be retrieved using available data, for instance, with the help of the Materials Project^40^). A possible tool that can be employed for the construction of perovskite slabs could be ASE software.^41^

**Figure 1:**
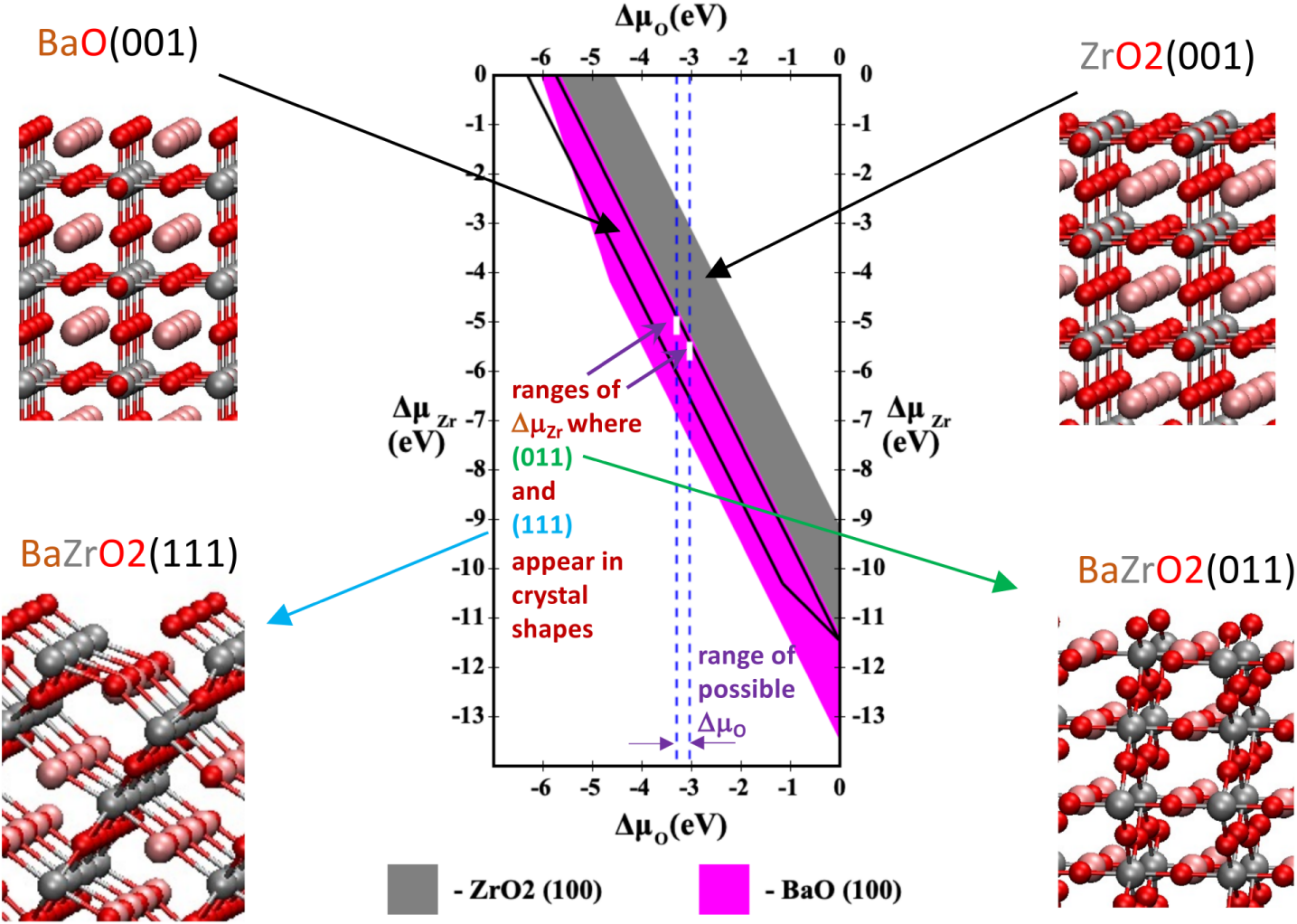
The surface phase diagram of BaZrO_3_. The studied terminations are presented too (left and right). The segments of stability of BaO and ZrO2 terminations of (001) surface are shown by pink and grey color, respectively. Stability wedge with respect to the precipitation of constituent oxides (BaO, BaO_2_ and ZrO_2_) is shown by black solid lines. The blue vertical lines confine the range of possible values of $\Delta \mu_{O}$ . The white lines highlight the values of $\Delta \mu_{Zr}$ , where (011) and (111) terminations could be observed in crystal shapes (see Figure 2).

According to Figure 1, we find that, in agreement with ref 12, BaO(001) and ZrO2(001) are two stable terminations in their respective ranges of chemical potentials of O and Zr. Also, in agreement with ref 12, we find that BaO(001) is stable with respect to constituent oxide formation, whereas ZrO2(001) is unstable with respect to ZrO_2_ bulk precipitation. Thus, BaO(001) prevails as the only stable termination of BaZrO_3_ in full agreement with ref 12.

We find that (011) and (111) terminations (slab stoichiometry subject to eqs 7 and 8) are not introduced into the phase diagram due to their higher free energies as compared with those of (001) terminations for all possible chemical potentials of O and Zr. However, in spite of the higher free energies of (011) and (111) terminations, these can still be introduced into the crystal shapes in view of their higher Miller indices compared to (001).

The free energy γ_*hkl*_ is a function of two chemical potentials (more precisely, their variations Δ*μ*_*Zr*_ and Δ*μ*_*O*_ in our case), and therefore, it will differ for their various acceptable values. Δ*μ*_*O*_ is confined to the range defined by the temperatures at operating/synthesis conditions (400–700 °C in a hydrogen atmosphere). The values of Δ*μ*_*Zr*_ should be confined to the stability wedge corresponding to the absence of precipitation of constituent oxides (black lines in Figures 1 and 6). Moreover, γ_*hkl*_ of (011) and (111) terminations should also be small enough to appear in a crystal shape (according to eq 6). In Figure 1, the ranges of acceptable Δ*μ*_*Zr*_ where (011) and (111) appear in crystal shapes are highlighted by the vertical white solid lines

We find that the γ_011_/γ_001_ and γ_111_/γ_001_ ratios are smaller for higher Δ*μ*_*Zr*_; therefore, the relative percentages of (011) and (111) terminations (vs (001)) in crystal shapes are higher for Δ*μ*_*Zr*_ at the upper end of the white lines in Figure 1. On the other hand, (001) termination prevails for low values of Δ*μ*_*Zr*_. In Figure 2, we display the crystal shapes at Δ*μ*_*O*_ = −3.28 eV (high temperatures) for values of Δ*μ*_*Zr*_ that provide the highest percentage of all three studied terminations.

**Figure 2:**
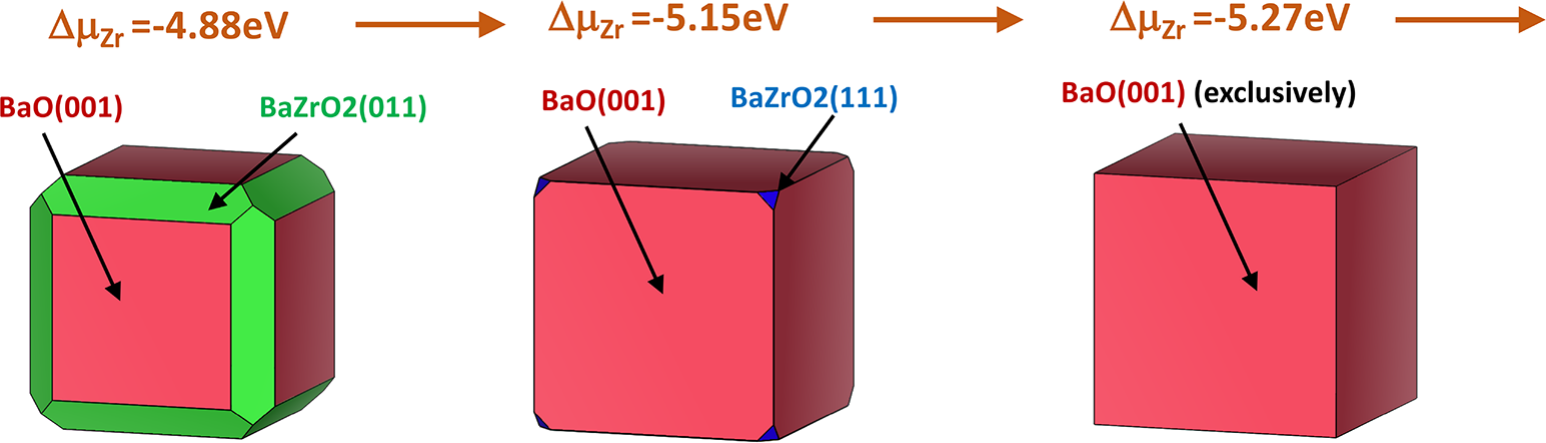
Crystal shapes of BaZrO_3_ at various values of Δ*μ_Zr_* (Δ*μ*_*O*_ = −3.28 eV). At higher values of Δ*μ_Zr_*, (011) and (111) terminations are introduced in the crystal shapes. At low Δ*μ*_*Zr*_, only the (001) termination prevails.

Figure 2 shows that (011) terminations could be present at a considerable and non-negligible percentage when Δ*μ*_*Zr*_ adopts higher values, which corresponds to Zr abundance (e.g., at synthesis conditions). In contrast, a very low percentage of (111) termination could be introduced at an intermediate value of Δ*μ*_*Zr*_, as shown in Figure 2. Overall, we find that although (001) terminations generally prevail, the (011) termination could also be introduced, particularly in the abundance of Zr, which corresponds to relatively high Δ*μ*_*Zr*_.

In Figure 2, we provided the crystal shapes for Δ*μ*_*O*_ = −3.28 eV. However, identical shapes could be constructed for another possible Δ*μ*_*O*_, equal to −3.04 eV (which corresponds to the lowest temperature of PCFC operation of 400 °C). In this case, the values of Δ*μ*_*Zr*_ should simply be decreased by a constant shift of 0.52 eV for each crystal shape shown in Figure 2. A more complex dependence of crystal shapes from Δ*μ*_*O*_ and Δ*μ*_*Zr*_ could also be possible, for instance, for Ni/BaZrO_3_ as we discuss in subsequent sections.

#### 3.1.2 Electronic and Structural Properties of BaZrO_3_ Surfaces

We also wish to elucidate possible reasons for the different stability of various terminations of BaZrO_3_, particularly stoichiometric and combined stoichiometric slabs (defined by eqs 7 and 8). Based on the values of surface free energies in the stability wedge with respect to constituent oxide precipitation (covered by the black solid lines in Figure 1), we find that stability is higher for terminations with lower Miller indices. This relative stability could be denoted as (001) > (011) > (111).

In Figure 3, we show the partial density of states (PDOS) of oxygen atoms in the outermost surface layer and two deeper subsurface layers for the slabs with all three types of Miller indices. For the least stable (111) termination, Figure 3a shows that the edges of high-energy peaks of occupied *p*-bands of oxygens of different near-surface layers are shifted upward for atoms located closer to the surface. This contrasts with the more stable (011) and (001) terminations, where all three edges are generally aligned (particularly for the most stable (001)). An upshift of the edges of the *p*-band of the surface oxygens (relative to the bulk) is an indicator of a weaker binding of the surface atoms, which possibly accounts for the lower stability of the (111) termination.

**Figure 3:**
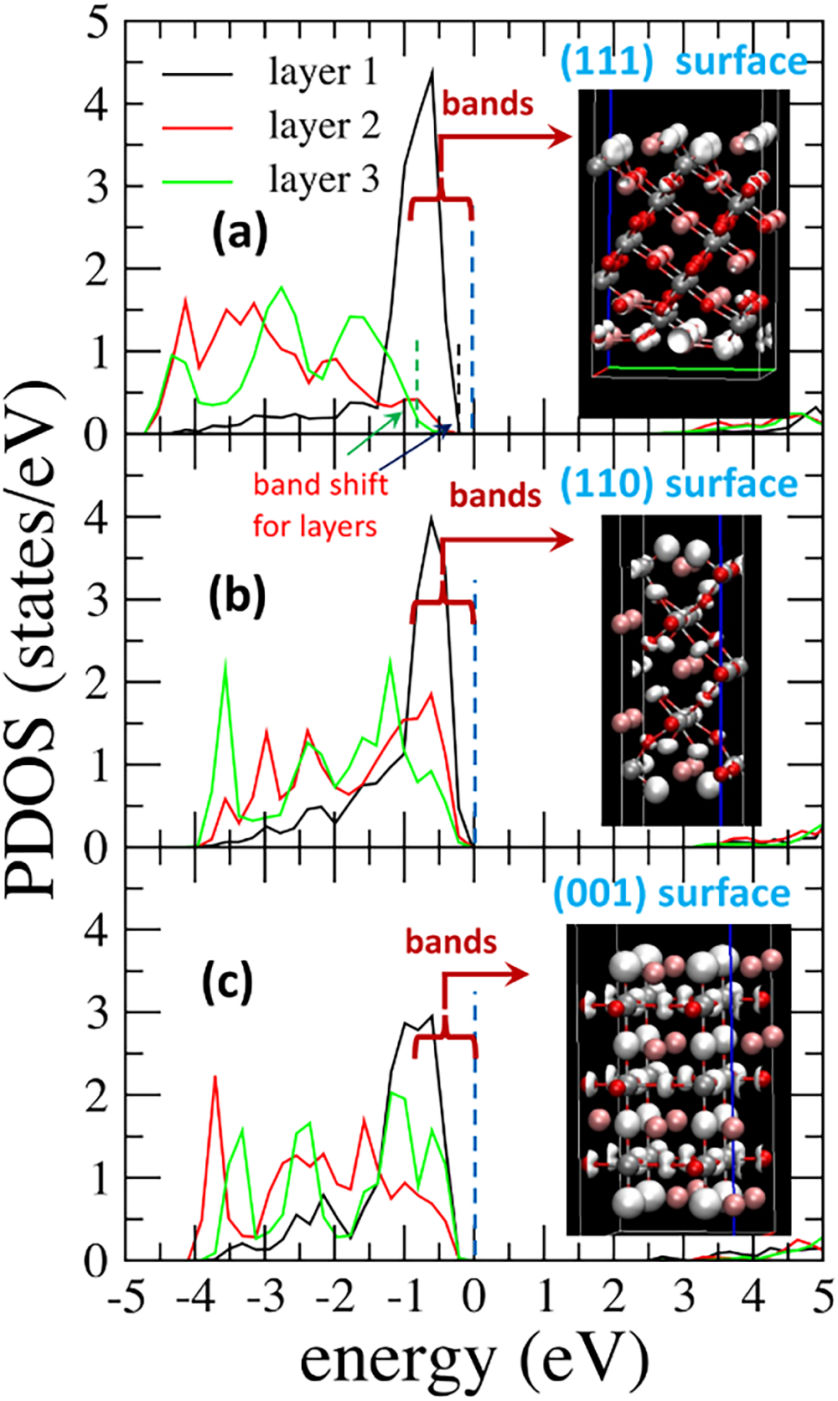
PDOS of *p*-states of oxygens in the surface and subsurface layers of (111), (011), and (001) terminations (a, b, c respectively, in the figure). Slabs are subject to eqs 7 or 8. The charge distributions of *p*-states, localized on oxygens in the energy range (from −1 to 0 eV vs Fermi level), are depicted on the right.

In Figure 3, we also provide the charge distribution of states, which span the energy range from −1 to 0 eV relative to the Fermi level (the PARCHG file, generated with the help of the LPARD flag, has been used for this purpose). Particularly for the (111) termination, the charge distribution of the states in question is clearly localized near the surface, whereas for the (011) and (001) slabs, this charge is more diffuse. The strong localization of the near-Fermi-level states exclusively on the surface of the (111) slab is also an indicator of weaker bonding of surface atoms.

To further elaborate on the underlying reasons for the different stabilities of the three studied terminations, we provide the data on local bonding and atomic charges of surface and subsurface Zr and Ba atoms in Table 1. The coordination numbers of Zr and Ba, defined as the numbers of neighboring atoms surrounding respective Zr and Ba, clearly indicate that generally higher values for Zr and Ba surface atoms correspond to more stable terminations. A low coordination number leads to shorter average bond lengths (due to stronger binding to neighboring oxygens) and a higher charge of *d*-states localized on Zr, as compared to the surface Zr of more stable terminations (*d* charges on ions of calculated systems are supplied as final results of VASP calculations).

**Table 1:** Coordination Numbers, Averaged Bond Lengths, and Charges Due to *d*-States for Ba and Zr Surface Atoms (First Values)^a^

surface	Zr coord. number	Ba coord. number	Zr–O bond length (Å)	Ba–O bond length (Å)	Zr *d*-electron charge	Ba *d*-electron charge
BaO(001)	6/6	8/12	2.122/2.123	2.945/3.009	1.416/1.38	0.26/0.248
ZrO2(001)	5/6	12/12	2.099/2.125	2.988/2.999	1.427/1.369	0.269/0.252
BaZrO2(011)	6/6	7/12	2.09/2.124	2.85/2.937	1.575/1.384	0.298/0.281
BaZrO2(111)	4/6	9/12	2.003/2.123	2.948/2.944	1.754/1.377	0.275/0.275

a The subsurface values (passed slash) are provided for comparison.

The relatively low coordination number of surface Zr atoms in BaZrO2(111) could be a factor that accounts for the low stability of this termination. The intermediate stability of BaZrO2(011) could be possibly explained by the low coordination of surface Ba. A low number of Ba–O bonds for the surface Ba of the (011) surface results in a high charge of *d*-states localized on the Ba. Thus, we find that smaller coordination numbers, particularly of surface Zr, are responsible for the lower stability of respective terminations.

### 3.2 Analysis of Ni/BaZrO_3_ Structure

#### 3.2.1 Effects of Ni on the Stability of BaZrO_3_ Terminations

Ni clusters, bound to BaZrO_3_ surfaces, could affect the stability of these perovskite terminations by altering their surface free energies. In this work, we employ the prismatic shape model of a Ni cluster (18 Ni atoms), previously used by one of us for the studies of Ni/YSZ and Ni/CeO_2_ interfaces.^42–45^ Within this model, the Ni cluster faces the gas phase with the low-energy Ni(111) termination.

In Section 3.1.1, we have indicated that although (011) and (111) have higher free energy than (001) terminations, such terminations, particularly (011), could be introduced into the phase diagram upon further stabilization (this is attested by the relatively high percentage of (011) terminations in crystal shape, e.g., at high values of possible Δ*μ*_*Zr*_, Figure 2). The important factor that could lead to the stabilization of stoichiometric (011) and possibly (111) terminations upon Ni addition is the introduction of more oxygens to the Ni/BaZrO_3_ interface. Although the introduction of more oxygens to the surface makes the slab nonstoichiometric (eqs 7 or 8 are not satisfied), the capture of these oxygens at the Ni/BaZrO_3_ interface is more favorable than on a pure BaZrO_3_ surface, which is caused by a charge transfer from Ni to the perovskite. Such stabilization of additional oxygens at the metal/oxide interface has been reported and discussed previously, for instance, for Ni/YSZ systems.^42,43,45^

We also need to state that, in this work, we limit the analysis of Ni impact on surface stabilization to the (001) and (011) terminations. The reason for this is the numerically close horizontal cell dimensions of the respective slabs, which allow us to employ identical models of Ni clusters, necessary for the analysis of Ni adsorption on the perovskite surface (the details of the formalism are discussed below). An additional aspect is also related to the analysis of the effect of extra oxygens introduced into the Ni/BaZrO_3_ interface, which also relies on the same model of surface Ni. This is not straightforward to add to the (111) termination, where the respective slab has incompatible in-plane dimensions.

In Figure 4, we provide the models of Ni/BaZrO_3_ interfaces where Ni clusters are mounted on BaO(001) and BaZrO2(011) terminations (left side of Figure 4; perovskite slabs are subject to eqs. 7 and 8). Upon structural optimization (structures presented on the left of Figure 4), we also studied oxygen introduction at the boundary between Ni and perovskite (Figure 4, right side, optimized structures). For both types of BaZrO_3_ terminations, the binding of oxygen is enhanced by the Ni cluster due to the charge transfer from Ni to perovskite slabs, measured as a depletion of the total charge of the Ni cluster. Using Bader’s approach, we find that for the Ni/BaO(001) interface, the charge of 1.05 e per each introduced oxygen is transferred from Ni to the perovskite, whereas for Ni/BaZrO(011), the charge of 0.95 e is removed from Ni upon the addition of one oxygen at the interface. In Figure 5, we provide the binding energies of oxygens (total number *n*) at two studied interfaces, defined as

**Figure 4:**
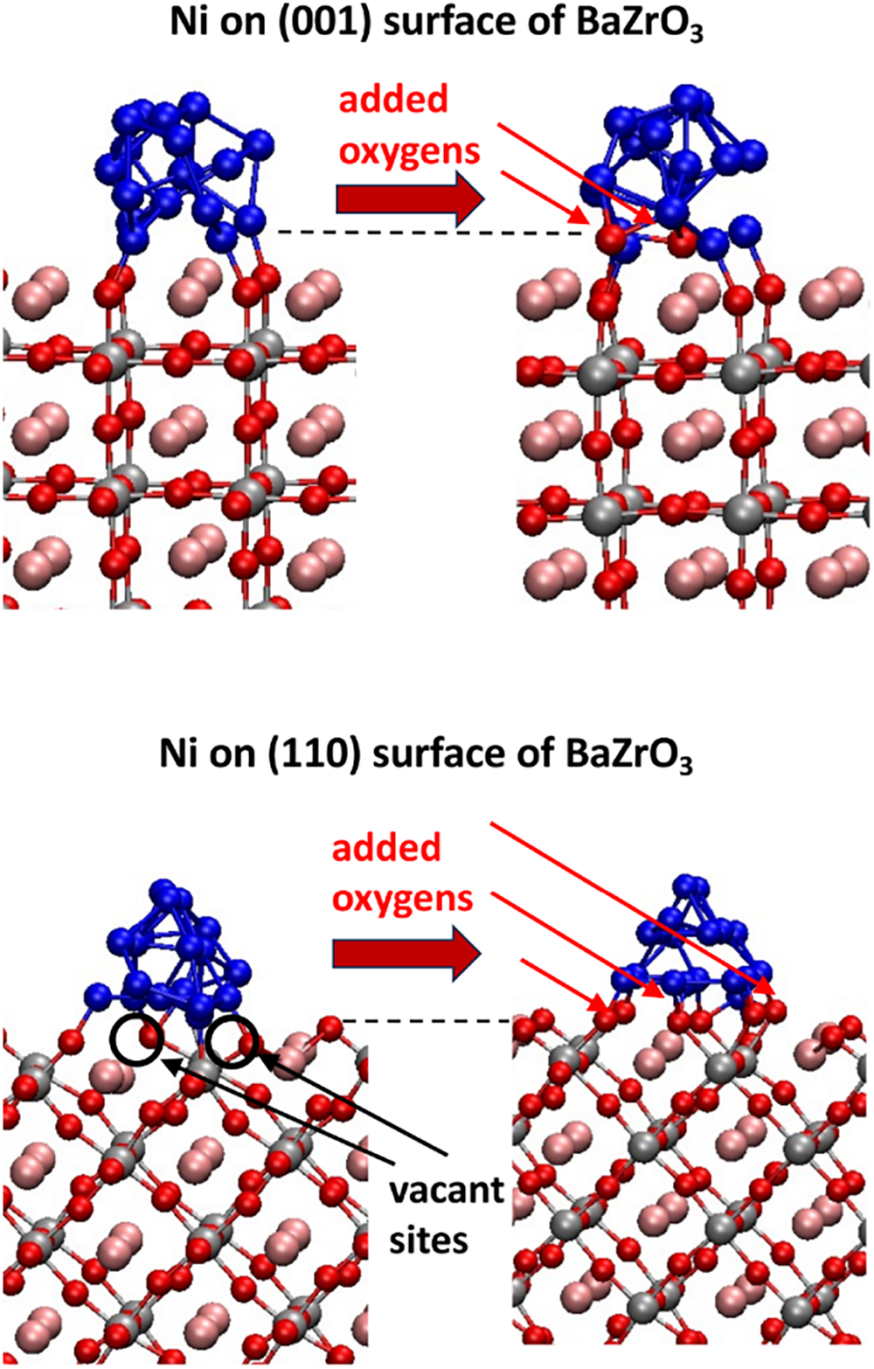
Models of Ni/BaZrO_3_ for BaO(001) and BaZrO2(011) terminations. Left side: optimized models prior to the introduction of additional oxygen. Right side: optimized models upon the introduction of additional oxygens. Broken horizontal lines highlight the planes where additional oxygens are introduced (in optimized structures).

**Figure 5:**
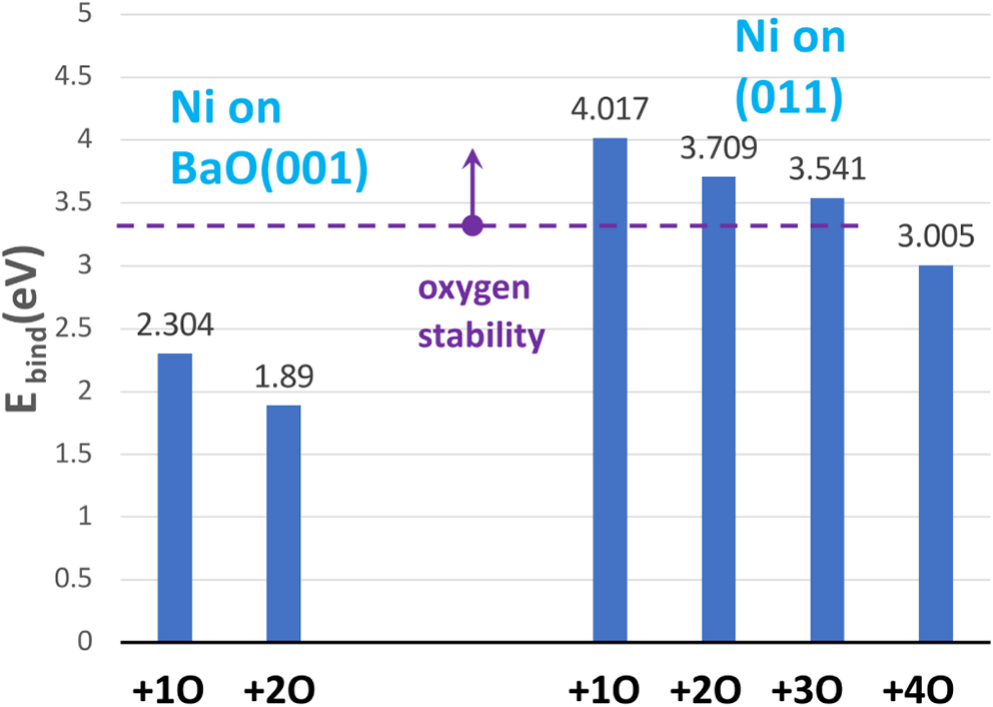
Binding energies of added oxygens at Ni/BaO(001) and Ni/BaZrO2(011) interfaces (calculated using eq 9). The boundary of the range of *E_bind_* where added oxygens are stable at synthesis conditions of Ni/BaZrO_3_ ^9^ is shown by the blue broken line.

9 $$E_{bind}=-\frac{1}{n}\left[E\left(\mathrm{Ni}/O_{3}+n\times O\right)-E(\mathrm{Ni}/{\mathrm{BaZrO}}_{3}\right)-0.5n\times h\left(O_{2}\right)]$$

where *h*(*O*_2_) is the energy of oxygen molecule (the empirically corrected value). The minimum binding energy required for stability of the added oxygen in a hydrogen atmosphere at the synthesis temperature^9^ is shown by the blue broken line, whereas the range of *E*_*bind*_ sufficient for oxygen stability is indicated by the vertical arrow. In spite of stabilization of captured oxygen by Ni in both studied systems, Figure 5 shows that capture of oxygens by Ni/BaZrO2(011) is significantly more favorable than by Ni/BaO(001). In fact, the extra oxygens at the Ni/BaZrO2(011) interface are stable under Ni/BaZrO_3_ synthesis conditions (the respective values are generally above the blue vertical line in Figure 5), whereas oxygen introduction at Ni/BaO(001) is not favorable energetically. To provide a possible explanation of a stronger binding of oxygens at the Ni/BaZrO2(011) interface, we analyzed structural properties of the two studied models.

In Figure 4, we show that additional oxygens have to be placed above the BaO plane of the respective (001) termination (top, right). Thus, additional oxygen atoms are introduced into the space between Ni and BaO, causing strong distortions in both structures. In contrast, for BaZrO2(011), the additional oxygens are introduced into the outermost BaZrO_2_ layer, filling the empty crystal sites (denoted as open circles). These sites are empty to account for eq 7; however, they could be filled if a stabilizing Ni cluster is introduced. The structural distortions of a lower degree (as compared to the Ni/BaO(001) interface) are introduced in this case, providing an explanation for the stronger binding of oxygen at the Ni/BaZrO2(011) interface.

One could notice that the binding energy per oxygen atom in Figure 5 decreases when more oxygens are introduced, although the same amount of charge is transferred from Ni to BaZrO_3_ for each added oxygen. This tendency of weakening oxygen binding could be explained by the shrinking of the offset between the top of the energy of valence bands localized on Ni and on BaZrO_3_ when more oxygens are added, as revealed by our calculations. This trend results in a less favorable binding of oxygens due to a smaller energy shift upon electron transfer from Ni to BaZrO_3_. We should note that the lowering of binding energy with the number of introduced additional oxygens is dependent on the model of the added Ni cluster, particularly its size. For a larger Ni cluster, the effect of electron transfer to BaZrO_3_ upon oxygen addition will be smaller. Thus, it is expected that the energy of oxygen binding would drop more rapidly with the number of added oxygens for larger Ni clusters. This aspect is critical for the analysis of the impact of Ni on perovskite surface stability.

#### 3.2.2 Phase Diagram and Crystal Shapes of Ni/BaZrO_3_

The free energy of a BaZrO_3_ surface with a mounted Ni cluster should, in principle, be expressed. However, herein we choose to estimate the difference of surface free energies instead. The binding of Ni to the BaZrO_3_ surface could be calculated as

10 $$E_{Ni/binding}=E(\mathrm{Ni}/{\mathrm{BaZrO}}_{3}+n\times O)-E({\mathrm{BaZrO}}_{3})-E\left(\mathrm{Ni}\right)$$

In eq 10, the actual stoichiometry of the BaZrO_3_ slab is not specified and is presented in a general form. As shown in Figure 5, for the model with BaO(001) termination, we find that oxygen is not stable with respect to scavenging by hydrogen at synthesis and PCFC operating temperatures (about 700 °C).^9^ Therefore, no extra oxygens are introduced when Ni is added on top of the BaO(001) surface. On the contrary, for the BaZrO2(011) termination, the addition of oxygen is stable with respect to scavenging by hydrogen, which is caused by a higher binding energy as compared to the Ni/BaO(001) interface.

For this reason, we evaluate the binding energy of Ni as a function of the number of oxygens (*n*), added at the interface for the Ni/BaZrO2(011) system:

11 $$E_{\mathrm{Ni}/binding}\left(n\right)=E\left(\mathrm{Ni}/{\mathrm{BaZrO}}_{2}\left(011\right)+n\times O\right)-E\left({\mathrm{BaZrO}}_{2}\left(011\right)\right)-E\left(\mathrm{Ni}\right)$$

The difference between the binding energies for two terminations (BaO(001) and BaZrO2(011)) is defined as

12 $$\Delta E_{Ni/binding}\left(n\right)=E_{Ni/binding}\left(\mathrm{Ni}/{\mathrm{BaZrO}}_{2}\left(011\right)+n\times O\right)-E_{Ni/binding}\left(\mathrm{Ni}/\mathrm{BaO}\left(001\right)\right)$$

To construct the free energy of BaZrO2(011) with mounted Ni and additional oxygens introduced at the interface, we added the Δ*E*_*Ni/biniding*_ and other additional terms to the expression of the free energy of BaZrO2(011) without the Ni cluster:

13 $$\gamma_{(011)+Ni}=\gamma_{\left(011\right)}+\alpha \left(\Delta E_{Ni/binding}-n\times \frac{1}{2}h\left(O_{2}\right)-n\times \Delta \mu_{O}\right)$$

Coefficient α in eq 13 is equal to the ratio of the surface areas of two employed slabs (for Ni/BaZrO_2_ and Ni/BaO). In this work, we also assume an equilibrium between Ni particles in the gas phase and the BaO(001) termination. This means that *E*_*Ni/biniding*_ for BaO(001) (eq 10) is equal to zero. This assumption allows us to assume that the relative stability (surface energy difference) of ZrO_2_(001) versus BaO(001) termination with an added Ni cluster is the same as for pure surfaces, which is justifiable in view of the weaker binding of Ni to ZrO2(001) as compared to BaO(001), according to our calculations.

Based on the introduced assumptions, we constructed the phase diagram for BaZrO_3_ terminations with an added Ni cluster, as shown in Figure 6. Figure 6 shows that BaZrO2(011) emerges on the phase diagram upon stabilization by Ni as is evident from the introduction of the green segment. The impact of added oxygen is described in Figure 6, where we provide the percentage of extra oxygen, assuming that 100% corresponds to the complete filling of all available vacant sites situated below the introduced Ni cluster. A greater percentage of extra oxygen results in a larger segment of BaZrO2(011) on the constructed diagrams (green area in Figure 6), which is expected due to greater stabilization by Ni. Complementary to the phase diagram, Figure 7 provides the crystal shapes for possible values of Δ*μ*_*O*_ (defined by the operating temperatures of PCFC) and values of Δ*μ*_*Zr*_, where (011) terminations are possible in crystal shapes.

**Figure 6:**
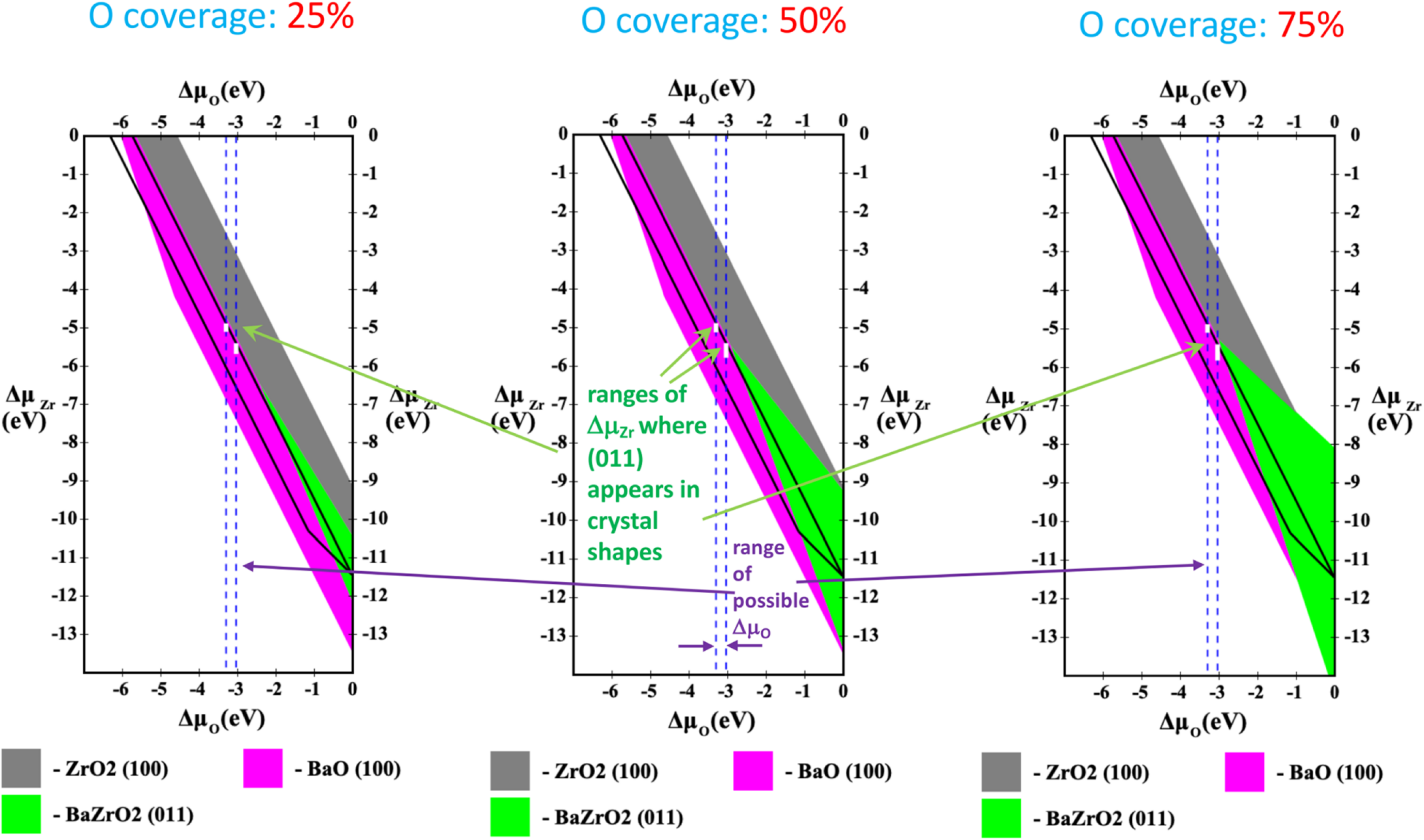
Phase diagrams for Ni/BaZrO_3_ with various percentages of extra oxygens added at the interface. The segments of stability of BaO and ZrO2 terminations of the (001) surface are shown in pink and gray, respectively. The stability wedge with respect to the precipitation of constituent oxides (BaO, BaO_2_, and ZrO_2_) is shown by black solid lines. The blue vertical lines confine the range of possible values of Δ*μ*_*O*_. The white lines highlight the values of Δ*μ*_*Zr*_, where (011) terminations could be observed in crystal shapes (see Figure 7).

**Figure 7:**
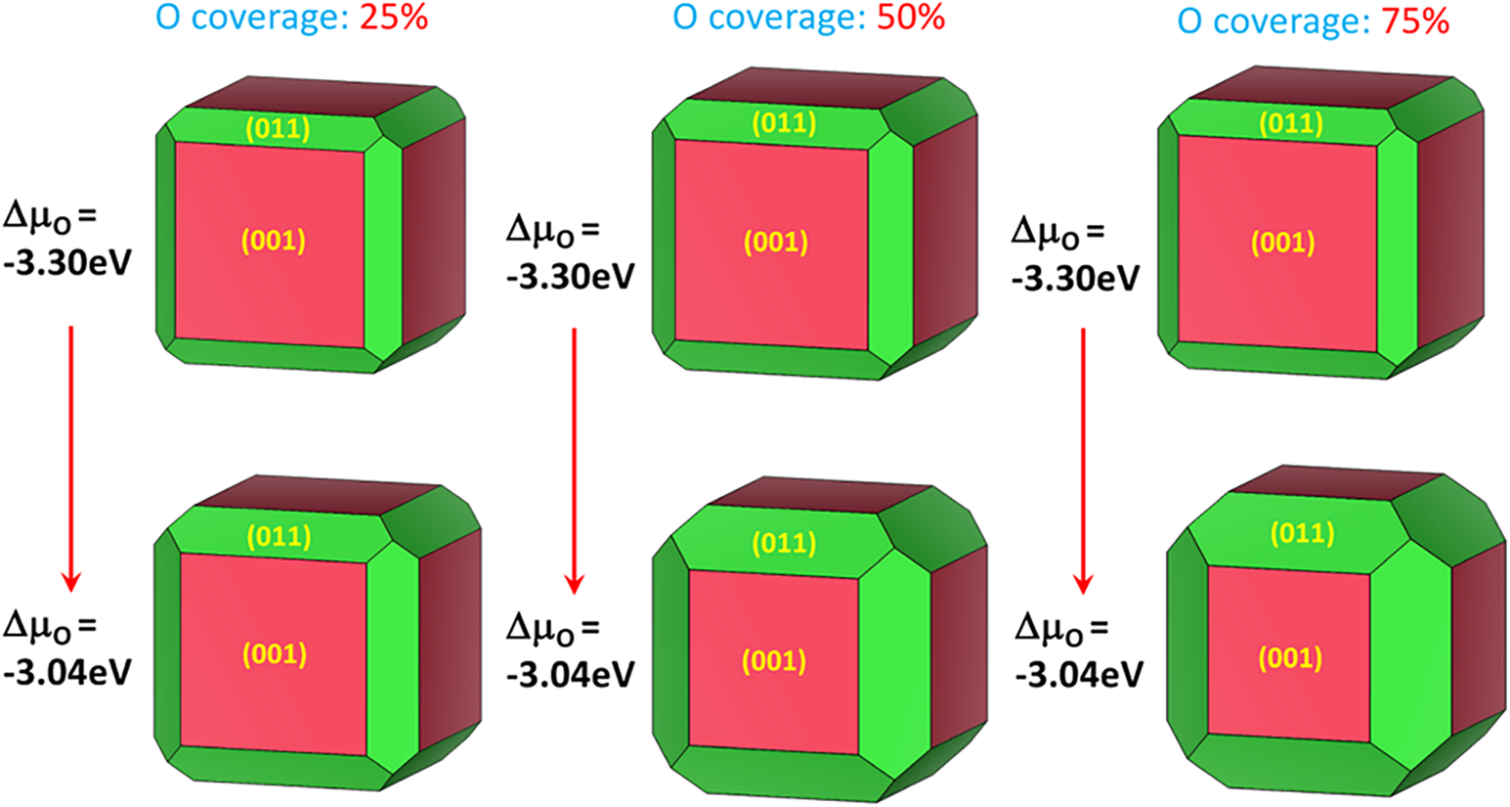
Crystal shapes of Ni/BaZrO_3_. The results for the highest possible Δ*μ*_*Zr*_ (top end of white lines in Figure 6) are provided. The change of the shape upon Δ*μ*_*Zr*_ increase (from −3.28 to −3.04 eV) is demonstrated for three types of oxygen coverages in the Ni/BaZrO_3_ interface.

Figure 7 shows that at low Δ*μ*_*O*_ values (high temperature 700 °C) the percentage of (011) terminations is relatively low, although not negligibly small. In fact, this percentage is similar to that for BaZrO_3_ without added Ni (see Figure 2). This could be explained by the relatively low energy of oxygen in the gas phase at the high temperature of 700 °C. At a higher chemical potential of Δ*μ*_*O*_ = −3.04 eV, (011) terminations are more strongly stabilized due to additional oxygen at the Ni/BaZrO_3_ interface.

Our results are also in general agreement with experimental studies (as discussed in the Introduction), where both {001} and {011} terminations have been observed^20,21^ subject to synthesis conditions and the types of Zr-containing precursors. We have shown that Ni clusters could stabilize the {011} termination, giving rise to its greater presence in BaZrO_3_ crystal shapes. Also, in agreement with experimental findings,^20^ our work shows that a higher chemical potential of Zr (Zr abundance) leads to a greater percentage of {011} terminations.

#### 3.2.3 Effect of Doping on the Relative Stability of Surface Terminations

Doping of BaZrO_3_, e.g., by Y, is usually introduced for hydrogen diffusion enhancement.^46,47^ Particularly, Y doping causes high proton conductivity of BaZrO_3_ and excellent stability in H_2_O and CO_2_ environment.^48^ Y doping also results in the formation of vacancies in BaZrO_3_ to account for charge neutrality. These vacancies could be filled by oxygen atoms, including the sites near the interface with Ni. Similar to extra oxygens introduced on the BaZrO2(011) surface at the interface with Ni, the extra oxygens added into the vacant sites introduced due to Y doping are also stabilized by electron transfer from Ni. Moreover, upon doping, the vacancies could also be formed on the BaO(001) termination, allowing for a stronger binding of additional oxygens at the interface with Ni as compared to the nondoped case. Figure 8 shows that additional oxygens could be added to the BaO(001) termination, unlike the nondoped case (shown in Figure 4), where extra oxygens are introduced between BaO(001) and the layer of Ni atoms. In this work, we introduced Y only to the surfaces of BaZrO_3_ to estimate the effect of extra oxygen capture at the interface, avoiding a more detailed description of Y distribution in the BaZrO_3_ bulk.

**Figure 8:**
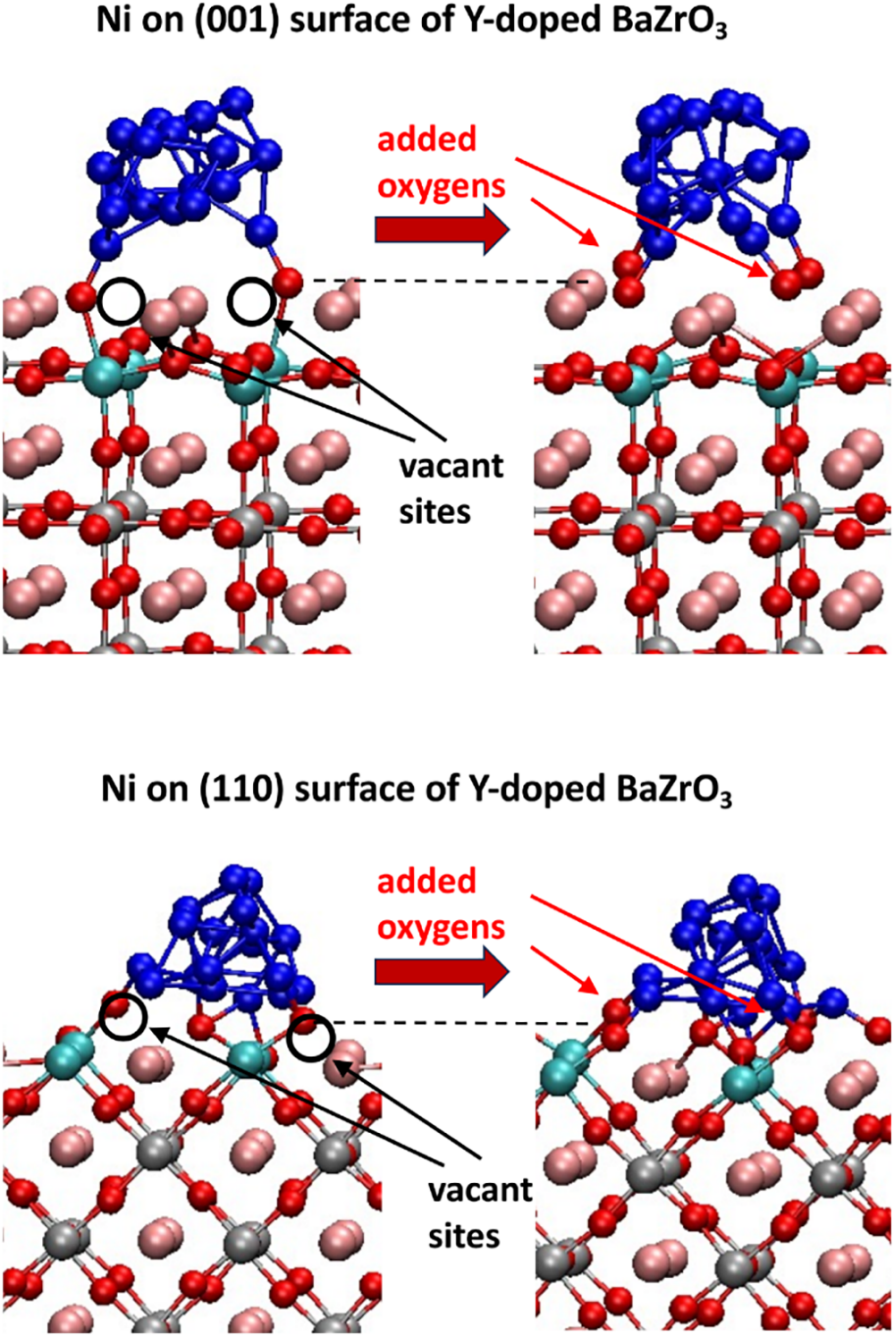
Models of Ni/Y-doped BaZrO_3_. Oxygens are added to the BaO(001) termination at the vacant sites. The added Y dopants are indicated by cyan color.

Figure 9 provides the oxygen binding energies for the two studied interfaces (according to eq 9). The key difference compared to the case of an interface between Ni and stoichiometric perovskite is the higher binding energies for the Ni/Y-doped BaO(001) termination. This is not unexpected, as the introduced additional oxygens cause much lower distortions in the surrounding environment (Ni and perovskites). For instance, the extra oxygens are not pushed into the space between Ni and the BaO(001) termination but instead reside within the BaO(001) plane.

**Figure 9:**
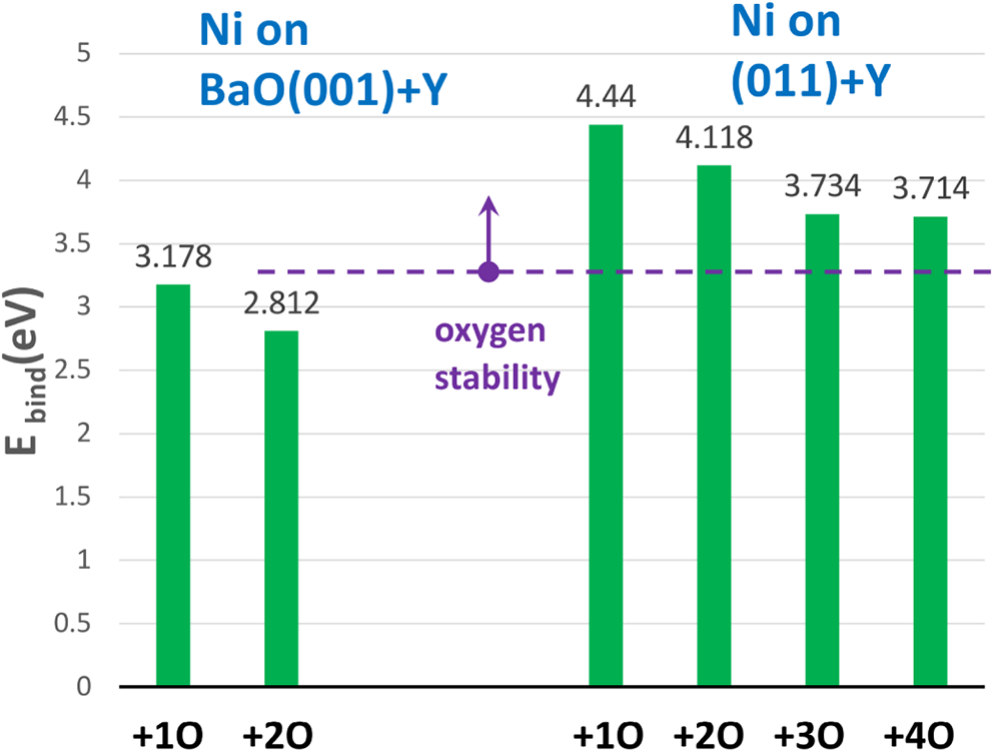
Binding energies of oxygens at the Ni/Y-doped BaZrO_3_ (similar to the oxygen binding energies at the Ni/BaZrO_3_ interface, shown in Figure 5). The boundary of the range of *E_bind_* where added oxygens are stable at the synthesis conditions of Ni/BaZrO_3_ ^9^ is shown by the blue broken line.

Although doping leads to stronger binding of oxygen to the BaO(001) surface, we should point out that doping also introduces more sites on BaZrO2(011) that could be filled by extra oxygens. Therefore, we assume that Y-doped BaZrO2(011) is still more stabilized upon Ni addition as compared to doped BaO(001) (similar to the relative stability of these surfaces without doping). We need to note that our provided analysis is very simplified, and a more thorough study of the atomic structure of the Ni/Y-doped BaZrO_3_ interface would be required to estimate the actual energies of oxygen incorporation into the metal/perovskite interfaces. At present, however, such a study would not be feasible in view of the absence of detailed information about atomic models of Ni/doped BaZrO_3_ interfaces. Indeed, even the structure of doped perovskites is still under debate,^49^ and thus, more efforts will be required in the future for obtaining detailed data on more complex Ni/doped BaZrO_3_ interfaces. Such a challenge is caused by a complex distribution of dopants near the surface and added Ni, which should be taken into account for the estimation of the energies of stabilization of the studied interfaces. In this work, we only make an observation of the enhanced binding of both studied terminations by Ni, assuming that the free energy difference could be approximated as equal to the case of Ni interfaces with nondoped surfaces. This assumption would allow us to construct models of stable Ni/doped BaZrO_3_ interfaces by simply introducing Y into the Ni/BaZrO_3_ models with undoped perovskite.

## 4 Conclusions

In this work, we provided an analysis of the surface stability of BaZrO_3_ and Ni/BaZrO_3_ structures. In line with previous reports, we find that BaO(001) is the termination featured by the lowest free energy, which is also stable with respect to constituent oxide formation.^12^ Although BaZrO2(011) has higher energy as compared to BaO or ZrO_2_ (in respective ranges of chemical potentials), this termination is present in crystal shapes and thus might appear in a synthesized material, particularly for high chemical potential of Zr. We should note that a relatively low percentage of BaZrO2(011) is predicted to be introduced, and BaO(001) should dominate.

We also provided an explanation as to why the slabs with three studied surface terminations (stoichiometric or combined stoichiometric, eqs 7 and 8) have increasing stability for surfaces with lower Miller indices ((111) < (011) < (001)). We attributed the higher stability of {001} terminations to a greater coordination number of Zr and Ba surface atoms. Lower coordination numbers of Ba on (011) and of Zr on (111) result in more pronounced distortions, such as shorter average bond lengths (as compared to those of {001} terminations). The surface distortions also result in the introduction of high-energy surface-localized states on the (011) and even more so on the (111) surface, whereas no strongly localized surface states are present for {001} terminations.

Introduction of Ni stabilizes both BaO(001) and BaZrO2(011) terminations; however, in the case of BaZrO2(011), additional oxygens can be added to the vacant sites, decreasing the free energy of this termination more than that of BaO(001), where the addition of extra oxygen causes stronger distortions. For this reason, the BaZrO2(011) termination is introduced into the phase diagram, in contrast to the case when Ni is not added to the perovskite. In crystal shapes, we find that Ni addition results in an increase of the BaZrO2(011) surface area due to stabilization caused by the incorporation of extra oxygens into the Ni/BaZrO2(011) interface. Introduction of the BaZrO2(011) termination via stabilization by a Ni cluster is in general agreement with experimental observations of such (011) terminations, presumably stabilized by Cl, N, and CH_3_ species.^20,21^ Our findings allow us to state that the percentage of (011) terminations could be increased via choosing lower temperatures (under operating and synthesis conditions) and/or upon abundance of Zr during synthesis, which corresponds to higher values of Zr chemical potential.

We have also evaluated the impact of doping on the binding of Ni to BaZrO_3_ surfaces. Due to vacancies introduced to sustain the charge neutrality of pure BaZrO_3_, both types of terminations (BaO and BaZrO_2_) could be further stabilized via the introduction of oxygen at the interface. The possible complex models of Ni/Y-doped BaZrO_3_, well-grounded on experimental and/or theoretical findings, are not established to date. Therefore, we rely on a simple assumption that the relative stability of BaZrO_2_ vs BaO termination in the doped structures is the same as in Ni/undoped BaZrO_3_. This assumption is based on our observation of the general stabilization of both surfaces of doped BaZrO_3_, thus keeping the difference between their free energies the same as that for the undoped interfaces.

Overall, our paper shows that, for the study of reactions at the Ni/BaZrO_3_ interface, one should, in principle, consider two types of models: Ni/BaO(001) and Ni/BaZrO2(011), with Y doping introduced into the perovskite. The constructed models could be employed for such future works.

## Supplementary Material



## Notes

Corrected energy of oxygen molecule, the estimates of oxygen chemical potential at PCFC operating temperatures (PDF)
