# Supplementary material for: The Role of Ni in Stabilizing BaZrO3 Surfaces at Ni/BaZrO3 Interfaces: A Density Functional Theory Analysis
Source: Langmuir. 2026 Jul 8;42(28):20093–103. doi: 10.1021/acs.langmuir.5c06802 (PMC13394392; doi:10.1021/acs.langmuir.5c06802)
Supplement: Supplementary file 1 [file la5c06802_si_001.pdf]

Supporting Information: The role of Ni in stabilizing the $BaZrO_3$ surfaces of Ni/$BaZrO_3$ interfaces: Density Functional Theory analysis

Maxim Shishkin* and Atsushi Ishikawa*

Department of Transdisciplinary Science and Engineering, School of Environment and Society, Institute of Science Tokyo, 2-12-1 Ookayama, Meguro-ku, Tokyo 152-8552, Japan

E-mail: shishkin.m.aa@m.titech.ac.jp; ishikawa.a.ai@m.titech.ac.jp

**SI. No.**	**Contents**	**Pg. No.**
1.	Evaluation of corrected energy of oxygen molecule	S-3
2.	Evaluation of oxygen chemical potential at PCFC operating temperatures	S-5
3.	References	S-6

S1. Evaluation of corrected energy of oxygen molecule

The total energy of an oxygen molecule in the triplet state, calculated with the help of PBE functional should be corrected for a good agreement of computed enthalpy of studied oxide formation with experiment. In this work we choose monoclinic phase of zirconia for adjustment of a total energy of O_2. The enthalpy of formation, denoted as ΔH is evaluated as:

$$\Delta H = h(Zr) - h(O_2) - h(ZrO_2) \tag{1}$$

Relying on the enthalpies of $h(Zr)$, $h(O_2)$ and $h(ZrO_2)$, calculated using PBE functional the underestimated value of ΔH is obtained due to a poor description of electronic structure of O_2 by PBE (similar to ref. [S1], we approximate that enthalpies at zero temperature are equal to total energies). For this reason instead of a total energy of O_2, determined by PBE (-9.86eV), the corrected value of -8.78eV should be used. The corrected $h(O_2)$ provides a good agreement of calculated ΔH as compared to experiment, whereas $h(Zr)$ and $h(ZrO_2)$ are determined by PBE calculations.

We have also estimated the energies of ZrO_2 and BaO formation using the hybrid HSE06 functional for comparison with PBE results. In Table S1 the deviations of energies of formation per atom in both formula units (ZrO_2 and BaO) for both employed functionals (PBE and HSE06) are provided (these are defined as the differences between ΔH, calculated according to eq.(1) and available experimental values divided by the number of atoms per unit).

Table S1 also lists the deviations of energies of formation per atom for corrected values of oxygen atom energies, chosen so that the calculated formation energy of one of the oxides would exactly match the experimental value, thus resulting in a zero deviation from experimental data (the energy of formation for the other oxide is not zero and provided for these cases in Table S1).

Table S1: Deviations of energies of oxide formation per atom (for ZrO_2 and BaO) from experiment obtained using PBE and HSE06 functionals (in eV units). The experimental values have been borrowed from external sources, e.g. ref. [S1].

Type of fitting	PBE		HSE06	
	$\Delta H(ZrO_2)$	$\Delta H(BaO)$	$\Delta H(ZrO_2)$	$\Delta H(BaO)$
no fitting	-0.366	-0.392	-0.178	-0.331
fitting of $\Delta H(ZrO_2)$	0	-0.121	0	-0.206
fitting of $\Delta H(BaO)$	0.159	0	0.266	0

According to Table S1, the energies of formation of both oxides are calculated in a closer agreement with experiment when HSE06 functional is employed. However, the enhanced accuracy of HSE06 calculations is still insufficient and further correction of oxygen energy would be required similar to the case of PBE calculations. Table S1 shows that upon correction, PBE-based absolute values are smaller (indicating a closer agreement with experimental data) as compared to HSE-based results. This poorer agreement for corrected HSE-based values stems from a better improvement of the energy of formation for ZrO_2 as compared to that of BaO when HSE is employed. For this reason, adjusted energy of an oxygen results in a greater deviation of HSE-based deviations as compared to corrected PBE-based values in Table S1.

In view of a better agreement of PBE-based corrected energies of formation for two studied oxides with experimental data we choose to employ this functional for analysis of stability of perovskite services. The PBE calculations are also more preferential due to much lower computational cost as compared to hybrid HSE06 approach.

S2. Evaluation of oxygen chemical potential at PCFC operating temperatures

For evaluation of oxygen chemical potential in hydrogen atmosphere the enthalpy of water formation, defined as:

$$\Delta H = h(H_2O) - h(H_2) - \frac{1}{2}h(O_2) \tag{2}$$

should be determined (where $h(H_2O)$, $h(H_2)$ are approximated to be equal to the total energies of respective species (ref. [S1]), whereas $h(O_2)$ is a a corrected enthalpy, determined by DFT). Using eq. (2), the variation of oxygen chemical potential could be calculated as:

$$\Delta\mu_O = \Delta H - T(S(H_2O) - S(H_2)) \tag{3}$$

where $S(H_2O)$ and $S(H_2)$ are the entropies of H_2O and H_2 respectively and T is the temperature. In view of an empirical correction of total energy of O_2 in this work we rely on empirical enthalpy of water formation, rather than computed value. The following empirical data is used in our work:

$$\begin{aligned} &\Delta H = -2.52eV; \\ &S(H_2O) = 1.97eV/(1000K); \\ &S(H_2) = 1.19eV/(1000K) \end{aligned} \tag{4}$$

The above data is taken from NIST database. We rely on the values of operating temperatures of PCFC equal to 400 and 700 °C. Simple calculations provide values of $\Delta\mu_O$ equal to 3.04eV and 3.28eV for 673 and 973K respectively.

References

(S1) Heifets, E.; Ho, J.; Merinov, B. Density functional simulation of the $BaZrO_3(011)$ surface structure. *Phys. Rev. B* **2007**, *75*, 155431.
